# ‘Et tu, inhibitor?’: the potential for HIV inhibitors to prime P-gp-mediated chemoresistance in cancer

**DOI:** 10.4155/fsoa-2017-0134

**Published:** 2017-11-23

**Authors:** Austin Y Shull, Christopher L Farrell

**Affiliations:** 1 Department of Biology, Presbyterian College, Clinton, SC 29325, USA; 2 Department of Pharmaceutical & Administrative Sciences, Presbyterian College School of Pharmacy, Clinton, SC 29325, USA

**Keywords:** cancer, chemoresistance, drug resistance, HIV inhibitors, P-gp, pharmacogenomics

The major hurdle regarding effective cancer treatment is the ability to prevent and overcome the mechanisms that drive chemoresistance. It is through chemoresistance that tumor populations are given opportunity to acquire more aggressive, metastatic phenotypes and decrease the chances of overall survival in patients carrying the pathophysiological burden of chemoresistant tumors. Based on this foundational issue regarding chemoresistance and cancer severity, efforts within the research community have been directed toward elucidating the molecular mechanisms that facilitate chemoresistance, many of which work in concert with one another. These chemoresistant mechanisms include treatment-based clonal selection, therapy-resistant somatic mutation development, induction of autophagy-mediated survival, increased expression of DNA repair enzymes and apoptotic resistance proteins, bypass signaling events via cross-talk with secondary extracellular signaling pathways and induction of multidrug resistance (MDR) efflux transporters [1]. While understanding of chemoresistant mechanisms has greatly expanded in recent years, one of the first chemoresistance mechanisms studied extensively, drug-induced overexpression of the MDR transporter P-gp, still remains unclear regarding the various ways in which it can be induced [2].

P-gp, encoded by the *ABCB1* gene, is a part of a superfamily of ABC transporters that expend ATP to facilitate transport of xenobiotic and metabolite substrates. First discovered in 1976, P-gp is found expressed in several tissue types within the body including the intestinal lumen, liver, kidney, brain and lymphocytic cells [3,4]. The physiological role of P-gp is to serve as an ATPase-mediated efflux pump of potentially toxic substrates to maintain intracellular homeostasis. Based on its established mechanism, activity of ABC transporters like P-gp have been important considerations when defining the pharmacokinetic and pharmacodynamic properties of therapeutic compounds [5]. This consideration for P-gp is especially true when it comes to administering chemotherapies that are known P-gp substrates. Such chemotherapies like vinblastine, etoposide, paclitaxel and doxorubicin are known P-gp substrates, and the wide-ranging activity of P-gp in cancer tissue types can dynamically affect the efficacy of these commonly administered chemotherapies [2]. This mechanistic role of P-gp has been especially critical when comparing naive tumor populations and chemotherapy-treated tumor populations who demonstrate an increase in P-gp expression when placed under the selective pressure of particular cancer therapy regimens. This established phenomenon is believed to be driven by multiple measures including the acquired induction of P-gp activity in the bulk tumor population as well as through chemotherapy-driven clonal selection and expansion of tumor populations that express higher levels of P-gp. In fact, recent reports have established connections between increased P-gp activity and cancer stem cells, which are known subpopulations of cancer cells involved in cancer progression and implicated in clonal selection-mediated chemoresistance [6–9]. In either case, P-gp induction plays a critical role in creating a chemoresistant phenotype in cancer cells, ultimately providing motivation for determining how to best mitigate the counteracting mechanisms of P-gp in cancer treatment.

One of the conventional directions taken when investigating P-gp-mediated resistance is understanding which chemotherapies induce this increased state of P-gp activity and determining how certain P-gp substrate chemotherapies could be co-administered rationally with compounds that counteract P-gp activity. Based on this direction, several classes of drug compounds have been identified as potential inhibitors of P-gp. Interestingly, one class of compounds identified to influence P-gp activity are established tyrosine-kinase inhibitors (TKIs) used in several cancer therapy regimens. While several studies report acquired resistance of single-agent TKI treatment being mediated through P-gp, several reports also reveal the ability for TKIs to counteract P-gp-mediated chemoresistance and increase the cytotoxic effects of P-gp substrate chemotherapies [10]. Such examples of TKIs demonstrating this inhibitory capability have been the BCR/ABL-targeting nilotonib [11], the VEGFR-targeting apatanib [12] and the EGFR-targeting lapatinib, gefitinib and erlonitib [13]. Determining how certain TKIs interact with P-gp could provide a double-edged therapeutic benefit against malignant progression based on both the canonical actions of TKIs disrupting the extracellular signaling cascade utilized for cancer growth as well as its noncanonical action of increasing intracellular accumulation of chemotherapies through the blockade of P-gp efflux action.

Nevertheless, while great efforts have been placed into better understanding the means for which to overcome P-gp resistance, little is still understood regarding the factors that make certain tumor populations more inclined to acquire a P-gp chemoresistant phenotype. Traditionally, studies that have investigated this mechanistic question employ a limited scope regarding the therapeutic agents that can increase P-gp activity in cancers, with most studies focusing on chemotherapies specifically and how they mediate an increase in P-gp expression and activity. However, with a reported count of over 300 compounds that are predicted to directly interact with P-gp, it can be reasonably posited that cancer chemotherapies are not the only class of pharmaceutical compounds that could manipulate the activity of P-gp in cancer cells [2,3]. This idea of nonchemotherapy-driven induction of cancer chemoresistance was first broached by a study from Lucia *et al*. who reported induced upregulation of P-gp expression in Kaposi's sarcoma cells through prolonged exposure of known HIV protease inhibitors including indinavir, nelfinavir and ritonavir. This upregulation in P-gp expression, seen at both the transcriptional and translational levels, was coupled with an increase in both doxorubicin and paclitaxel resistance in each of the prolonged HIV protease inhibitor-treated SLK Kaposi's sarcoma cell lines as well as an increased efflux of the fluorescent dye rhodamine 123, which is used to measure P-gp-mediated efflux. These results are quite intriguing based on the notion that the nonchemotherapeutic P-gp substrates of these HIV protease inhibitors could induce a chemotherapy-resistant phenotype *in vitro*. Interestingly, the means in which the HIV protease inhibitors induce these phenotypes are perhaps different from the means in which P-gp substrate chemotherapies induce a P-gp based on the observation that doxorubicin could increase P-gp expression within 72 h, whereas prolonged HIV protease inhibitor treatment took approximately 6 months to demonstrate prevalent expression of P-gp. These results suggest that while P-gp substrate chemotherapies like doxorubicin or paclitaxel can induce acute expression of P-gp, prolonged exposure of nonchemotherapy P-gp substrates like HIV protease inhibitors perhaps provide a selective pressure where cells with higher P-gp levels would be given a selective advantage during cancer propagation and/or tissue renewal [14].

While results observed by Lucia *et al*. demonstrate the ability of HIV drugs to induce increased expression of P-gp within *in vitro* cell lines, a report from Kis *et al*. demonstrated similar correlation between P-gp expression and prolonged antiretroviral therapy (ART) treatment based on interrogation of intestinal samples from ART-treated HIV patients. By performing microarray-based differential expression analysis between healthy controls, HIV patients with no prior administration of ARTs, and HIV patients who have been on a recurrent regimen of ARTs for >1 year, Kis *et al*. determined and validated through RT-PCR, western blot and immunohistochemistry that P-gp expression increased significantly in the intestinal tissue of HIV patients treated long term with ART regimens when compared with both the naive HIV patient and healthy donor cohorts [15]. Thus, these results of nonchemotherapy driven induction of P-gp expression within clinical intestinal samples complement results observed within *in vitro* cell lines that were under the selective pressure of HIV protease inhibitors.

Nonetheless, while the increased P-gp expression demonstrated by Kis *et al*. was observed in noncancerous intestinal tissue, the coinciding results of Lucia *et al*. provide consideration of whether induction of P-gp through prolonged treatment of nonchemotherapeutic compounds could prime certain tissue types to be less sensitive to chemotherapy treatments if cancer were to arise within the respective tissue. This phenomenon could very well be feasible specifically for colon tissue based on the dynamics of colon cancer development and the opportunity for drug compounds like HIV inhibitors to establish increased expression of P-gp. Thus, by coalescing the established information of P-gp-driven MDR in cancer and recent reports demonstrating the ability of nonchemotherapeutic inhibitors of HIV to increase P-gp activity in both cancerous and noncancerous tissue, future direction should be considered in determining which commonly administered drug regimens have the potential to prime cancer-susceptible tissues to be more resistant to chemotherapies that are P-gp substrates. This question is especially prevalent based on recent studies that report a significant increase in prescription use among the adult population in the USA as well a significant increase in medication use among adults 65 years or older in England [16,17]. Thus, investigating commonly prescribed medications and their potential to selectively increase P-gp activity in tissue that are susceptible to cancer initiation, tumorigenic progression and chemoresistance should be intentionally considered.
